# Associations between bacterial infections and blood pressure in pregnancy

**DOI:** 10.1016/j.preghy.2017.09.004

**Published:** 2017-10

**Authors:** Clive J. Petry, Ken K. Ong, Ieuan A. Hughes, Carlo L. Acerini, David B. Dunger

**Affiliations:** a Department of Paediatrics, University of Cambridge, Cambridge, UK; b Medical Research Council Epidemiology Unit, University of Cambridge, Cambridge, UK; c The Institute of Metabolic Science, University of Cambridge, Cambridge, UK

**Keywords:** Gestational hypertension, Pre-eclampsia, Antibiotics, Pregnancy

## Abstract

**Objectives:**

To test the hypothesis that bacterial infections in pregnancy are related to maternal blood pressure.

**Study design:**

Bacterial infection was assessed using antibiotic usage as a surrogate and its association with blood pressure in pregnancy tested in the Cambridge Baby Growth Study.

**Main outcome measures:**

Antibiotic usage in pregnancy was self-reported in questionnaires. Blood pressure measurements at four time points in pregnancy were collected from the hospital notes of 622 women.

**Results:**

Using all the available blood pressure readings (adjusted for weeks gestation) antibiotic usage was associated with a higher mean arterial blood pressure across pregnancy: antibiotics used 85 (84, 87) mmHg vs. no antibiotics used 83 (83, 84) mmHg (β = 2.3 (0.6, 4.0) mmHg, p = 9.6 × 10^−3^, from 621 individuals). Further analysis revealed that antibiotic usage was associated with diastolic (β = 2.3 (0.6, 4.0) mmHg; p = 7.0 × 10^−3^) more than systolic blood pressure (β = 1.4 (−0.9, 3.7) mmHg; p = 0.2). The effect size associated with antibiotic usage appeared to rise slightly after the first trimester.

**Conclusions:**

Bacterial infection in pregnancy, as assessed by self-reported antibiotic usage, is associated with small rises in blood pressure.

## Highlights

• Antibiotic use in pregnancy was associated with a 2–3 mmHg rise in blood pressure.
• It was related more to changes in diastolic than systolic blood pressure.
• The most likely cause is exposure to bacterial infections.

## 1 Introduction

Pre-eclampsia remains a leading cause of maternal and perinatal mortality and morbidity. Its established risk factors include null parity, maternal age >40 years, multiple pregnancy, extended times between pregnancies, the presence of antiphospholipid antibodies, prior pre-eclampsia in a previous pregnancy or a positive family history, chronic hypertension or gestational hypertension during pregnancy, pre-gestational or gestational diabetes, obesity and use of assisted reproductive technology [1], [2]. Less established potential risk factors include infection [3] and consequences of infection such as inflammation. Indeed the usual inflammatory response observed in uneventful pregnancies is enhanced in pregnancies affected by pre-eclampsia [4]. It has been suggested that the link between infection and the development of pre-eclampsia could be at the level of its initiation, due to an increased risk of uteroplacental atherosis (fibrinoid necrosis of the vessel wall with subintimal accumulations of lipophages), and/or its progression, through an increase in the maternal inflammatory response during pregnancy [5].

A number of studies have sought associations between infections in pregnancy and the development of pre-eclampsia. Whilst not all studies have found significant associations [3], most have found a positive association, as supported by subsequent meta-analyses [6], [7]. Less is known about potential links between infections and less severe rises in blood pressure in pregnancy. To investigate this we studied antibiotic usage in pregnancy as a surrogate of bacterial infection exposure, plus urinary tract infection (UTI) exposure (the commonest form of bacterial infection in pregnancy), to test the hypothesis that bacterial infection in pregnancy is associated with rises in blood pressure.

## 2 Materials and methods

### 2.1 Cohort

The prospective and longitudinal Cambridge Baby Growth Study recruited 2229 mothers (and their partners and offspring) attending ultrasound clinics during early pregnancy at the Rosie Maternity Hospital, Cambridge, United Kingdom, between 2001 and 2009 [8]. All study participants were over 16 years of age and for this study, women who took anti-hypertensive drugs were excluded. Participants who may have had raised blood pressure at certain points during the pregnancy, e.g. during labour, but who did not report anti-hypertensive usage were still included in the study. Fasting blood samples were collected from 1239 participants for the measurement of plasma glucose and insulin concentrations around week 28 of pregnancy for the evaluation of insulin sensitivity by Homeostasis Model Assessment (HOMA) modelling [9]. In this cohort, 96.9% of the offspring were of white ethnicity, 0.8% were of mixed race, 0.6% were black (African or Caribbean), 0.8% were East-Asian, and 0.9% were Indo-Asian.

### 2.2 Antibiotic usage

Each of the study participants were given a printed questionnaire at recruitment to fill in and return once the pregnancy was completed [10]. One of the questions asked “Have you taken any medicine during this pregnancy?” Those women who responded in the affirmative were then asked to complete a table with the following headings: “Name”, “Disease”, “Daily Dose”, “No. of Days” and “Gestational Week(s)”. From these questionnaires drugs were categorised into three major dichotomous groups: paracetamol-containing drugs, drugs used to treat indigestion and antibiotics. No account was taken of the number of times that a particular drug was taken, the specific drug that was taken or the doses consumed.

For the purposes of this study only the category based on antibiotic usage was employed. The timings when the antibiotics were reported having been taken were divided into trimesters (first trimester being up to gestational week 12, second trimester being weeks 13–27 and third trimester being from week 28 onwards). Of the 1271 women that filled out questionnaires, 173 (13.6%) reported that they had taken antibiotics during pregnancy and 1098 had not. Of the women that reported having taken antibiotics in pregnancy 51 reported first trimester usage, 68 second trimester and 54 third trimester (some women did not specify the timing when they took antibiotics, whereas some of the others reported taking them in more than one trimester). Specific antibiotic usage was reported as follows: amoxycillin (71 women), cephalexin/cefalexin (16), penicillin (16), erythromycin (15), flucloxacillin (6), clarithromycin (3), augmentin (2), cephradine (2), ampicillin (1), cefaclor (1), cefotaxime (1), ciproflaxine (1), metronidazole (1), trimethoprim (1) and ‘antibiotic(s)’ (37).

### 2.3 Urinary tract infections

The most common reason given for taking antibiotics during pregnancy was to treat UTIs. Of the 1271 women that filled out their questionnaires, 53 (4.2%) self-reported that they had experienced UTIs at some point during their pregnancy. Of these women 19 reported having had UTIs in the first trimester of pregnancy, 25 in the second trimester and 14 in the third trimester; some women reported having had UTIs in more than one trimester and some did not disclose when in pregnancy they were infected.

### 2.4 Blood pressure during pregnancy

Routine blood pressure measurements during pregnancy that had been recorded in hospital notes were collected from a total of 968 women in the Cambridge Baby Growth Study (other hospital notes either not being available to us or the blood pressures not being recorded in the notes) [8]. They were grouped into one of four readings according to the gestational week at which the measurements were taken: (1) at 11.8 (11.5, 12.0) weeks, (2) at 31.4 (31.3, 31.5) weeks and (3) at 37.0 (36.9, 37.0) weeks. The fourth readings were taken during the final 2 weeks prior to parturition (mean 38.8 weeks), parturition occurring at 39.8 (39.7, 39.9) weeks. Blood pressure measurements were available from 622 women for whom self-reported antibiotic usage was available (84 (13.5%) of whom had taken antibiotics during pregnancy). The characteristics of those who we had blood pressure readings for, according to whether they took antibiotics or not are shown in Table 1. There were no significant differences between the groups, although the pre-pregnancy BMI was borderline higher in those who subsequently took antibiotics. In those women where we had blood pressure readings 27 (4.3%) reported that they had had at least one UTI during their pregnancy.

**Table 1:** Characteristics of those Cambridge Baby Growth Study participants who reported to have taken antibiotics during pregnancy and those that did not in women that we had blood pressure readings from.

Characteristic	Women who reported taking antibiotics during pregnancy (n = 84)	Women who did not report taking antibiotics during pregnancy (n = 538)	p-value
Maternal age (years)	33.4 (32.4, 34.4)	33.6 (33.3, 34.0)	0.7
Parity	1.7 (1.5, 1.9)	1.7 (1.6, 1.8)	1.0
Gestational age at baby’s birth (weeks)	39.8 (39.5, 40.1)	40.0 (39.8, 40.1)	0.4
Birth weight of baby (kg)	3.530 (3.433, 3.628)	3.479 (3.440, 3.518)	0.3
Percentage giving birth to males	51.8	51.3	0.9
Pre-pregnancy BMI (kg/m^2^)	24.7 (23.7, 25.6)	23.7 (23.3, 24.1)	0.06
Percentage that reported smoking	4.8	2.6	0.3
Percentage with gestational diabetes	11.7	10.9	0.9

The birth weights of the offspring were adjusted for gestational age at birth, sex, parity and maternal BMI before pregnancy.

### 2.5 Ethics

The Cambridge Baby Growth Study was approved by the local ethics committee, Addenbrooke’s Hospital, Cambridge, United Kingdom. All procedures followed were in accordance with the institutional guidelines. Written informed consent was obtained from all the study participants.

### 2.6 Assays

Blood glucose concentrations were measured using a routine glucose oxidase-based method. Plasma insulin concentrations were measured using a DSL ELISA kit (London, U.K.) according to the manufacturer’s instructions.

### 2.7 Calculations

Mean arterial blood pressure was estimated as twice the diastolic plus the systolic blood pressure all divided by three. The body mass index (BMI) before pregnancy was calculated as the pre-pregnancy body weight divided by the height squared. HOMA S was calculated using the online calculator available at https://www.dtu.ox.ac.uk/homacalculator/ [9].

### 2.8 Statistical analysis

The associations between antibiotic usage (or UTI) at any time during pregnancy and blood pressure (mean arterial, systolic or diastolic) were tested using general estimation equation modelling, adjusting for weeks of gestation (and sometimes BMI) when the blood pressure readings were taken. Associations at individual time points and between antibiotic use and HOMA S (insulin sensitivity) or between blood pressure and antibiotic use/UTIs in specific trimesters were assessed by linear regression. Values for those women that experienced UTIs were compared with those from women who did not report any antibiotic usage at all (those women who reported antibiotic usage but did not specify why they took antibiotics and those that reported other types of infection were removed from the analyses to avoid potential confounders). Unless otherwise stated all other data are presented as means (95% confidence intervals). Statistical analyses were performed using Stata 13 (StataCorp LP, College Station, Texas, U.S.A.). P < 0.05 was considered statistically significant throughout.

## 3 Results

### 3.1 Associations between antibiotic usage at any time during pregnancy and blood pressure

**Fig. 1:**
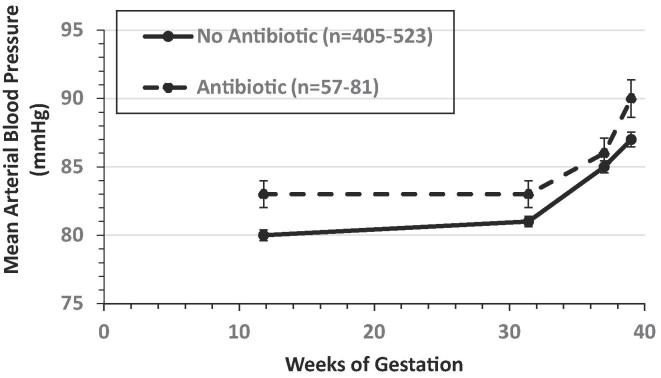
Mean arterial blood pressures in women that reported use of antibiotics during pregnancy and the women that did not in the Cambridge Baby Growth Study. Data are mean (S.E.M.).

Antibiotic usage was associated with a higher mean arterial blood pressure in pregnancy as a whole, mean arterial blood pressures over the four readings recorded during pregnancy being: antibiotics used 85 (84, 87) mmHg vs. no antibiotics used 83 (83, 84) mmHg (β = 2.3 (0.6, 4.0) mmHg, p = 9.6 × 10^−3^, from 621 individuals) (Fig. 1). Further adjustment for BMI did not appreciably change the outcome: antibiotics used 86 (84, 88) mmHg vs. no antibiotics used 83 (83, 84) mmHg (β = 2.7 (0.4, 5.0) mmHg, p = 0.02, from 434 individuals).

Separating the mean arterial blood pressure into its component systolic and diastolic parts, antibiotic usage was not associated with systolic blood pressures over the four readings taken over pregnancy: antibiotics used 115 (113, 117) mmHg vs. no antibiotics used 114 (113, 114) mmHg (β = 1.4 (−0.9, 3.7) mmHg, p = 0.2). Further adjustment for BMI led to even more overlap between the two groups: antibiotics used 114 (111, 117) mmHg vs. no antibiotics used 114 (113, 115) mmHg (β = −0.1 (−3.4, 3.3) mmHg, p = 1.0, from 434 individuals). Antibiotic usage, however, was associated with diastolic blood pressure over the same readings: antibiotics used 70 (69, 72) mmHg vs. no antibiotics used 68 (67, 69) mmHg (β = 2.3 (0.6, 4.0) mmHg, p = 7.0 × 10^−3^), although further adjustment for BMI weakened the relationship as the 95% confidence interval for the diastolic blood pressures of those women who used antibiotics widened: antibiotics used 70 (68, 73) mmHg vs. no antibiotics used 68 (67, 69) mmHg (β = 2.2 (−0.2, 4.6) mmHg, p = 0.08, from 434 individuals).

### 3.2 Associations between antibiotic usage in specific trimesters of pregnancy and blood pressure

Table 2 shows associations between antibiotic usage in specific trimesters of pregnancy and mean arterial blood pressures at time points close to those particular trimesters. Here the mean effect size appeared to rise after the first trimester.

**Table 2:** Mean arterial blood pressures and antibiotic usage in specific trimesters of pregnancy (using readings taken close to that specific trimester).

Gestation when blood pressure was recorded (weeks)	Trimester of pregnancy when antibiotics were reported as being used	Women who used antibiotics (mmHg)	Women who did not use antibiotics (mmHg)	β (mmHg)	p-value
11.8	1	80 (77, 84) (n = 29)	80 (79, 81) (n = 520)	0.2 (−3.1, 3.5)	0.9
11.8	2	82 (79, 85) (n = 34)	80 (79, 81) (n = 520)	1.8 (−1.2, 4.9)	0.2
31.4	2	84 (81, 87) (n = 34)	81 (80, 82) (n = 523)	2.5 (−0.5, 5.6)	0.1
31.4	3	84 (80, 88) (n = 19)	81 (80, 82) (n = 523)	3.2 (−0.8, 7.3)	0.1
37.0	3	87 (83, 92) (n = 17)	85 (84, 86) (n = 511)	2.7 (−2.0, 7.4)	0.3
38.8	3	89 (84, 95) (n = 16)	87 (86, 88) (n = 405)	2.7 (−2.5, 7.9)	0.3

The mean arterial blood pressures were adjusted for the number of weeks gestation when the measurements were taken throughout. Data are mean (95% confidence interval).

### 3.3 Associations with HOMA S (insulin sensitivity)

Each of the blood pressure readings were associated with reduced week 28 HOMA S: week 11.8 (β′ = −0.216, p = 2.7 × 10^−6^, n = 465), week 31.4 (β′ = −0.267, p = 4.3 × 10^−9^, n = 468), week 37.0 (β′ = −0.303, p = 2.7 × 10^−11^, n = 458) and week 38.8 (β′ = −0.228, p = 1.8 × 10^−5^, n = 348). However there was no association between antibiotic usage and HOMA S: antibiotics used 109.4 (99.6, 120.3) %S (n = 123) vs. no antibiotics used 109.6 (105.5, 113.8) %S (n = 751) (p = 1.0).

### 3.4 Associations with urinary tract infection(s)

The association between exposure to UTI(s) during pregnancy and overall mean arterial blood pressure was not statistically significant: UTI 85 (82, 88) mmHg (n = 27) vs. no antibiotic use 84 (84, 85) mmHg (n = 516) (β = 0.7 (−2.0, 3.4) mmHg, p = 0.6). Table 3 shows the comparison of individual mean arterial blood pressures between those women who experienced UTI(s) during pregnancy and those that did not. The effect sizes were positive but small and not statistically significant for all the blood pressure associations with UTIs.

**Table 3:** Maternal mean arterial blood pressures in pregnancy and in women who experienced UTI(s) during pregnancy compared with those without infection.

Gestation (weeks)	Experienced urinary tract infection(s) during pregnancy	No antibiotic usage during pregnancy	β (mmHg)	p-value
11.8	81 (77, 84) (n = 27)	80 (79, 81) (n = 514)	0.4 (−3.0, 3.8)	0.8
31.4	83 (79, 86) (n = 27)	81 (80, 82) (n = 516)	1.6 (−1.9, 5.0)	0.4
37.0	86 (82, 89) (n = 26)	85 (84, 86) (n = 506)	0.9 (−2.9, 4.8)	0.6
38.8	89 (84, 94) (n = 19)	87 (86, 88) (n = 402)	2.3 (−2.4, 7.1)	0.3

The mean arterial blood pressures were adjusted for the number of weeks gestation when the measurements were taken throughout. Data are mean (95% confidence interval).

## 4 Discussion

In this study we have shown that in our population self-reported general antibiotic usage in pregnancy is associated with a small increase in mean arterial blood pressure of around 2–3 mmHg. Although infections, such as those requiring treatment with antibiotics, have previously been shown to be associated with severe hypertensive disorders of pregnancy [6], [7], this is the first time that a surrogate of bacterial infections has been shown to be associated with raised blood pressure in pregnancy *per se*. This rise in blood pressure appeared to relate to the diastolic more than the systolic blood pressure measurements. Those women who reported that they took antibiotics had slightly higher pre-pregnancy BMIs than those that did not (there being a borderline statistical rather than a clinical difference) but adjusting for this did not change the outcome so the antibiotic-associated change in blood pressure did not relate directly to obesity.

Although there appears to be a link between infection and pre-eclampsia [6], [7] less has been reported about infection and less severe forms of pregnancy-induced hypertension. Two studies showed associations between UTIs and increased risk of gestational hypertension [11], [12] whereas another study reported a reduced frequency of non-proteinuric pregnancy-induced hypertension in women infected with *T. gondii* [13] (explained by long-term antibiotic usage reducing further bacterial infections). Based on these studies we believe that our association between raised blood pressure in pregnancy and antibiotic usage reflects exposure to bacterial infections of sufficient severity to merit treatment. Indeed when testing associations between maternal blood pressures at different time points across pregnancy and UTIs, we found effect sizes of 0.4–2.3 mmHg increases in mean arterial blood pressure in the infected group (albeit without statistical significance, presumably because of insufficient statistical power). We could not detect significant associations when testing specific trimesters of active infection, although again all the effect sizes were positive. The largest of these were in the third trimester of pregnancy, when the overall effect size associated with all women who took antibiotics during pregnancy was actually decreasing (Fig. 1). This was probably due to the effect of including blood pressures of women who used antibiotics earlier in pregnancy but who were free of infection by the third trimester.

There are a number of other possible explanations to explain the associations between antibiotic usage in pregnancy and increases in blood pressure, although they appear to be less convincing. Firstly antibiotics could have a direct effect on blood pressure in pregnancy, although we do not know of any published evidence of this. Secondly rises in blood pressure can sometimes be associated with effects of antibiotic allergy [14], although it seems unlikely to have affected our population, due to the infrequency of the allergic reaction causing increases in blood pressure [15] and the fact that at the ages of the women studied here most of them would probably have avoided taking antibiotics that they knew they were allergic to (especially in pregnancy). Thirdly some parenterally-administered antibiotic preparations contain sodium [16], intake of which could raise blood pressure. However none of the women studied indicated that they received antibiotics by any route other than orally. A fourth explanation is that antibiotics could indirectly influence the blood pressure through causing changes to the microbiome, as observed in rodent models [17], [18], [19]. Whilst this is potentially the most plausible alternative explanation for the association between antibiotic use in pregnancy and increases in blood pressure [20], there is no evidence for it in humans at present and in rodents changes to the microbiome sometimes lead to reductions rather than increases in blood pressure [19].

In our study the mechanism behind the raised blood pressure did not appear to involve altered insulin sensitivity, despite the established link between insulin resistance and hypertensive syndromes of pregnancy [21], [22]. One potential mechanism that is independent of changes in insulin sensitivity, however, involves altered endothelial nitric oxide synthase (eNOS) activity which regulates vascular tone. *In vitro* studies of human umbilical vein endothelial cells suggest that infectious stimuli are able to lower eNOS gene expression [23]. Consistent with this, serum nitric oxide concentrations appear to be reduced in pre-eclampsia [24]. Our association between antibiotic usage and rises in blood pressure relating more to diastolic rather than systolic blood pressure may also be consistent with this given that effects of changes in nitric oxide production would tend to affect arterial resistance rather than cardiac output, which comprises a greater part of the diastolic than the systolic blood pressure.

Although the results from this study are interesting it does have limitations. Firstly it appears underpowered to detect associations with UTIs, with antibiotic usage at specific time points and with specific antibiotic usage, especially when the overall rise in mean arterial blood pressure in women who took antibiotics in pregnancy was only 2–3 mmHg. Another study limitation is that self-reporting prescription drug intakes can be affected by recall bias, and recall for drugs with short-term use such as antibiotics may be lower than that for drugs used to treat chronic conditions [25]. However recall bias through forgetting antibiotic consumption would tend to reduce the effect size rather than increase it. As we found a significant association even with potential recall bias our conclusions would not change if the bias lessened however. We did not have continuous blood pressure readings from pregnancy and it is possibly that certain study participants may have had raised blood pressures at times not specifically analysed in this study, e.g. during labour. However unless there was a difference in the occurrence rates of such occasions between our groups, it is unlikely to have significantly affected our overall results. Finally it remains possible that the significant association arose due to either random variation or microbiome alterations. However given the established links between infection and pre-eclampsia [6], [7], [11], [12], [13] and the fact that there is a potential mechanism available, our interpretation of the primary association appears most convincing.

In summary we have shown for the first time that self-reported antibiotic use in pregnancy, most likely reflecting bacterial infections of sufficient severity to merit antibiotic treatment, is associated with a modest rise in mean arterial blood pressure. Partially consistent with our findings, in a study over 6000 (non-pregnant) women of all ages from the 1960s the mean systolic blood pressures of women with significant bacturia were around 3 mmHg higher than those without bacturia, although statistical significance was not reached on this occasion [26]. Our findings are therefore unlikely to have arisen by chance and although our detected effect size on blood pressure is small, the findings underpin published links between infection in pregnancy and pre-eclampsia.
